# Psilocybin for treatment resistant depression in patients taking a concomitant SSRI medication

**DOI:** 10.1038/s41386-023-01648-7

**Published:** 2023-07-13

**Authors:** Guy M. Goodwin, Megan Croal, David Feifel, John R. Kelly, Lindsey Marwood, Sunil Mistry, Veronica O’Keane, Stephanie Knatz Peck, Hollie Simmons, Claudia Sisa, Susan C. Stansfield, Joyce Tsai, Sam Williams, Ekaterina Malievskaia

**Affiliations:** 1 COMPASS Pathfinder Ltd, London, United Kingdom; 2 Kadima Neuropsychiatry Institute, San Diego, CA USA; 3 Department of Psychiatry, Trinity Centre for Health Sciences, Tallaght University Hospital, Dublin, Ireland; 4 Department of Psychiatry, University of California San Diego, San Diego, CA USA

**Keywords:** Drug development, Outcomes research

## Abstract

Psilocybin is being investigated as a treatment in adults with treatment-resistant depression (TRD). Withdrawal from serotonergic antidepressant drugs is a common prerequisite for taking part in trials of psilocybin due to the possibility of ongoing antidepressant drugs altering the psychedelic effect. This phase II, exploratory, international, fixed-dose, open-label study explored the safety, tolerability, and efficacy of a synthetic form of psilocybin (investigational drug COMP360) adjunct to a selective serotonin reuptake inhibitor in participants with TRD. Participants received a single 25 mg dose of psilocybin alongside psychological support and were followed-up for 3 weeks. The primary efficacy end point was change in the Montgomery-Åsberg Depression Rating Scale (MADRS) total score from Baseline at Week 3. Secondary end points were safety, including treatment-emergent adverse events (TEAEs), the proportion of responders and remitters at Week 3, and the change from Baseline to Week 3 in Clinical Global Impression–Severity (CGI-S) score. Nineteen participants were dosed and the mean Baseline MADRS total score was 31.7 (SD = 5.77). Twelve (63.2%) participants had a TEAE, most of which were mild and resolved on the day of onset. There were no serious TEAEs or indication of increased suicidal ideation or behavior. At Week 3, mean change from Baseline in MADRS total score was −14.9 (95% CI, −20.7 to −9.2), and −1.3 (SD = 1.29) in the CGI-S. Both response and remission were evident in 8 (42.1%) participants. Larger, comparator-controlled trials are necessary to understand if this paradigm can optimize treatment-outcome where antidepressant drug withdrawal would be problematic.

## Introduction

Psilocybin has demonstrated antidepressant properties in preclinical models and small trials of participants with cancer-related depression, major depressive disorder (MDD), and treatment-resistant depression (TRD) [1–4]. The therapeutic efficacy of a single of dose of psilocybin was recently supported in a randomized double-blind clinical trial of 233 participants with TRD, the largest such trial to date [5, 6]. Although the mechanism(s) through which acute psilocybin exerts its lasting antidepressant effect remain unclear, post-hoc analyses revealing correlations between the intensity of altered or mystical state of consciousness and therapeutic outcome suggest that the acute psychedelic experience may play an important role [7, 8].

Reports from surveys suggest that the acute subjective psychedelic effects of psilocybin, and of another serotonergic drug, lysergic acid diethylamide (LSD), are diminished by use of antidepressant drugs in the weeks or months prior [9–11]. More convincingly, chronic administration of antidepressant drugs, including a selective serotonin reuptake inhibitor (SSRI), reduced the head-twitch response, a stereotypic behavior exhibited by rodents in response to 5-HT2A agonizts and also downregulated 5-HT2A receptors, which are considered essential for the subjective psychedelic effects of psilocybin [12, 13]. Indeed, peak subjective psychedelic effects of psilocybin correlate with dose-dependent 5-HT2A receptor occupancy in the brain [14, 15]. Additionally, co-administration of 5-HT2A antagonist ketanserin has been reported to reduce the intensity of the subjective effects of several psychedelics in humans [16, 17]. Psilocin also weakly inhibits the serotonin transporter site that is the target for SSRIs [18]. Thus, the pharmacological effects of psilocybin could be attenuated by SSRIs either via an indirect effect such as downregulation of 5-HT receptors or a direct interaction with the transporter. In accordance with this literature, all previous trials of psilocybin in cancer patients, MDD, and TRD have withdrawn participants from antidepressant drugs prior to administering psilocybin [5, 19, 20].

In contrast, a recent double-blind randomized controlled trial in healthy participants found that administration of an SSRI for two weeks did not significantly alter the acute subjective effects of 25 mg of psilocybin [21] suggesting that the practice of discontinuing stable antidepressant drug treatment prior to initiating psilocybin therapy may unnecessarily add complexity to study procedures. Discontinuing antidepressant drugs prior to initiating psilocybin therapy presents challenges to clinical implementation, experimental design, and data interpretation, such as increasing the risk of adverse effects of withdrawal (which could additionally hinder data interpretation), excluding otherwise eligible participants from trials, and limiting future accessibility. With the development of psilocybin specifically as a treatment for TRD, it is important to test the impact of chronic antidepressant treatment on the antidepressant effects of psilocybin. This outcome will inform whether it is necessary to withdraw people from antidepressant drugs prior to administering psilocybin for a safe or therapeutic effect.

This exploratory phase II clinical trial used an open-label, fixed-dose design to investigate the safety and efficacy of a synthetic form of psilocybin with psychological support, adjunct to an SSRI, in participants experiencing a treatment-resistant episode of MDD.

## Materials and methods

### Trial oversight

This was a phase II, exploratory, fixed-dose, open-label study conducted between August 2020 and September 2021. This trial explored the safety and efficacy of a single dose of 25 mg of the investigational drug COMP360, a proprietary pharmaceutical-grade synthetic psilocybin formulation, optimized for stability and purity, adjunct to an antidepressant drug in participants experiencing a current episode of treatment-resistant MDD (EudraCT number: 2018 002377; Clinicaltrials.gov identifier: NCT04739865). COMP360 was supplied by the sponsor, COMPASS Pathfinder Ltd (a subsidiary of COMPASS Pathways plc), London, UK. The protocol was approved by ethics committees at two sites located in Ireland and the United States (US): the Clinical Research Ethics Committee of the Cork Teaching Hospitals (CREC), and the Advarra Center for IRB Intelligence (CIRBI). Participants provided written informed consent prior to participation.

The sponsor supervised the study which was conducted by a contract research organization (Worldwide Clinical Trials, Nottingham, UK, and North Carolina, US). The study was conducted in accordance with the International Conference on Harmonisation Good Clinical Practice guideline, and the ethical principles of the Declaration of Helsinki.

### Participants

This study recruited adults aged 18 years or older who were outpatients and referred by practitioners from specialized psychiatric services or word of mouth from primary care services. Participants were experiencing TRD, defined by meeting the Diagnostic and Statistical Manual of Mental Disorders (5^th^ edition) criteria of single or recurrent episode of MDD (if first single episode, duration >3 months ≤2 years was required) without psychotic features and with at least moderate symptom severity (Hamilton Depression Rating Scale, 17 item [HAM-D-17] >18) at Screening and Baseline, and having failed to respond to an adequate dose and duration (>8 weeks) of two to four pharmacological treatments for the current episode, including their current SSRI [22–24]. Participants entered this study having been previously pre-screened for our phase IIb clinical trial (COMP 001), which was not an open label study, and the current trial (COMP 003) simultaneously. In COMP 001, 428 patients were screened and 233 were found to be eligible and underwent randomization having successfully withdrawn from any antidepressant drug as required by the protocol. Patients unable or unwilling to complete medication withdrawal at the two participating sites, or those who had a preference to be screened for COMP 003 instead, were considered for this study.

Participants were taking a locally approved therapeutic dose of a single SSRI with at least 75% adherence for a minimum of six weeks prior to taking part and were asked to continue this treatment for the duration of the study. Permitted SSRIs included citalopram, escitalopram, fluoxetine, paroxetine, sertraline, vilazodone, and vortioxetine. Participants taking multiple antidepressant therapies were excluded.

Participants experiencing a major comorbid psychiatric disorder or suicide risk were excluded based on clinical assessment, medical records, the Mini International Neuropsychiatric Interview (version 7.0.2), the McLean screening instrument for borderline personality disorder, and the Columbia-Suicide Severity Rating Scale (C-SSRS) [25–27]. A full list of eligibility criteria and procedures are provided in the Supplementary Appendix.

### Study design and procedures

Eligible participants entered the screening period and attended the clinic weekly for three weeks prior to the psilocybin administration session. During this time, eligibility and safety assessments took place including monitoring of medication changes, suicidality (C-SSRS), electrocardiograms (ECGs), blood tests, and vital signs.

Preparation for the psilocybin experience was conducted by a therapist who was trained by the sponsor through a specially devised program [28]. All therapists were mental health professionals with relevant experience, training, and licensure to satisfy requirements and act in this capacity on the study. This included PhD clinical psychologists and other qualifications depending on the country. Preparation took place over three sessions, during which the therapist built trust with the participant, explained the trial design and procedures, provided psychoeducation, and helped to prepare the participant for the psilocybin experience. The final preparation session took place the day before administration (Baseline, Day -1), where safety measures were conducted to confirm continued eligibility, and participants underwent efficacy assessments. The Schedule of Assessments in the Supplementary Appendix (Supplementary Table S1) includes full details on the assessments conducted.

The administration session (Day 1) consisted of a single administration of psilocybin 25 mg which could be administered to up to six participants in different rooms simultaneously. Each participant was accompanied by a lead therapist, with whom they had completed preparation, for the six- to eight-hour session to ensure physical and psychological safety and encourage the participant to remain attentive to the natural unfolding of their subjective experience, while avoiding active guidance. An assisting therapist was available within the vicinity to step in at any time if the lead therapist needed to leave or additional support was required, and a study psychiatrist was on site. Blood pressure was monitored continuously using a finger cuff device. If simultaneous administration took place, the lead therapist remained with the participant and one assisting therapist was available by moving between rooms. Participants wore eyeshades and headphones with a specifically designed playlist of music to assist in directing their attention internally. Participants returned home after the acute drug effects had passed.

Participants were followed up for three weeks to monitor safety and efficacy. At Day 2 and Week 1 following administration, participants completed an integration session with their lead therapist who encouraged them to derive their own solutions and insights from the psilocybin experience.

### Safety end points

Safety outcomes included ECG, clinical laboratory tests, vital signs, incidence of adverse events, and change from Baseline in suicidal ideation and behavior measured by the C-SSRS.

Adverse events were examined at every visit and followed until resolved or stable. Treatment-emergent adverse events (TEAEs) were coded using the Medical Dictionary for Regulatory Activities version 23.0.

### Efficacy end points

The primary efficacy end point was the change in Montgomery-Åsberg Depression Rating Scale (MADRS) total score from Baseline (Day -1) to three weeks post-psilocybin administration (Week 3) [29]. Total scores in the MADRS range from 0-60, with higher scores indicating greater severity of depression. The MADRS was administered by a remote, blinded independent rater, following the structured interview guide [30] at Baseline (Day -1), Day 2 and Weeks 1, 2, and 3 to ensure standardization of administration and questions (service was provided by Worldwide Clinical Trials).

Key secondary end points included the proportion of participants with a response (defined as a ≥50% improvement in MADRS total score from Baseline) and remission (defined as a MADRS total score ≤10) at Week 3, change from Baseline in Clinical Global Impression–Severity (CGI-S) score at Week 3 and proportion of participants with a response (defined as a score on the CGI-S of 1 (“normal, not ill at all”) or 2 (“borderline mentally ill”)) [31]. The CGI-S is a 7-point rating scale of severity based on clinical judgment.

### Exploratory end points

Exploratory end points included change in total score from Baseline to Week 3 in participant EQ-5D-3 level (EQ-5D-3L) which captures an index of health state based on response to five dimensions of quality of life on a three-point scale ranging from “no problems” to “extreme problems” and additionally includes the EQ-VAS ranging from “worst imaginable health state” (score = 0) to “best imaginable health state” (score =100), Generalized Anxiety Disorder 7-item (GAD-7) where total scores range from 0-21 with higher scores indicating greater severity of anxiety, self-reported Quick Inventory of Depressive Symptomatology 16-item (QIDS-SR-16) where total scores range from 0-27 and higher scores indicate greater severity of depression, the Clinical Global Impression-Improvement (CGI-I) which is a 7-point rating scale of improvement based on clinical judgment, with lower scores indicating greater improvement [32–35], and the change from Baseline to Day 2 in Positive and Negative Affect Schedule (PANAS) where total scores range from 10-50 for both the positive and negative affect subscales [36].

The acute subjective psychedelic experience was captured by the Five-Dimensional Altered States of Consciousness questionnaire (5D-ASC) once the acute psychedelic effects had subsided (end of Day 1) [37–39]. The intensity of experience relating to each dimension of the 5D-ASC was captured on a VAS ranging from “No, not more than usually” (0 mm) to “Yes, much more than usually” (100 mm). Dimensions include visual restructuralization (perceptual and imaginational alterations such as visuals, illusions, or synesthesia’s), oceanic boundlessness (positive mystical-type experiences including derealization and depersonalization associated with positive emotional states, such as heightened mood, euphoric exaltation, and dissolution of time and space), reduction of vigilance (impaired alertness and clouded cognition or consciousness), anxious ego dissolution (negative dysphoric experiences associated with anxiety that arise from ego disintegration and loss of self-control phenomena) and auditory alterations (perceptions and acoustic hallucinations).

### Statistical analysis

No formal sample size calculations were performed for this study. There was no imputation for missing efficacy data and the observed data were used in statistical analyses.

Safety analyses were performed on the safety analysis set which included all participants who received psilocybin. Descriptive statistics were used to analyze safety data including TEAEs, concomitant treatments, evaluations of vital signs, clinical laboratory tests, findings from 12-lead ECGs, and suicidality assessments (C-SSRS).

Efficacy analyses were performed on the full analysis set which included all participants who received psilocybin and had at least one post-Baseline efficacy assessment. The primary efficacy end point (change from Baseline to Week 3 in the MADRS total score) was evaluated with the use of descriptive summaries using statistics for continuous variables. Similarly, the secondary and exploratory efficacy end points were also summarized descriptively.

## Results

### Participants

Nineteen out of 24 enrolled participants with TRD were dosed and completed the study - there were no post-treatment withdrawals. All enrolled participants were included in the safety and full analysis sets and one participant was excluded from the per- protocol analysis set due to violation of exclusion criterion two: Prior electroconvulsive therapy and/or ketamine for current episode (Supplementary Fig. S1).

The stable and ongoing SSRIs included sertraline, escitalopram, fluoxetine, vilazodone, paroxetine, and citalopram (Table 1). All participants continued their SSRI medication throughout the follow-up period and to the end of the study (Week 3); two participants on escitalopram increased their dose (10–20 mg) during the study. The mean duration of SSRI treatment prior to psilocybin administration (including changes in dose if treatment was continuous) was 14.68 months (SD = 13.213).

**Table 1:** Baseline demographic and clinical characteristics (safety analysis set).

Variable	COMP360 25 mg + SSRI (*N* = 19)
Demographics	
Female, *n* (%)	13 (68.4)
Age, years, mean (SD)	42.2 (10.80)
Race, White, *n* (%)	15 (78.9)
Body mass index, kg/m^2^, mean (SD)	26.26 (6.521)
Psychiatric history	
Duration of current depressive episode, *n* (%)	
<1 year	3 (15.8)
1 year to <2 years	13 (68.4)
>2 years	3 (15.8)
Failed treatments during the current depressive episode, *n* (%)	
2	12 (63.2)
3 or 4	7 (36.8)
Ongoing SSRI treatment, *n* (%) [min dose, max dose]	
Sertraline	6 (31.6) [50 mg, 200 mg]
Escitalopram	6 (31.6) [5 mg, 20 mg]
Fluoxetine	3 (15.8%) [20 mg, 80 mg]
Vilazodone	2 (10.5%) [20 mg, 40 mg]
Paroxetine	1 (5.3%) [60 mg]
Citalopram	1 (5.3%) [20 mg]
Duration of SSRI treatment prior to Baseline months mean (SD)^a^	14.68 (13.213)
Baseline depression scores	
MADRS total score	
Mean (SD)	31.7 (5.77)
Moderate (20–30), *n* (%)	7 (36.8)

*MADRS* Montgomery-Åsberg Depression Rating Scale, *SD* standard deviation, *SSRI* selective serotonin reuptake inhibitor.

^a^Previous doses of an SSRI contributed to the duration of treatment provided that the treatment was continuous with no breaks between doses.

The majority of participants were female (68.4%) and White (78.9%), and the mean age at Screening was 42.2 years (SD = 10.80). The length of current episode of depression was greater than one year for 84.2% of participants, and 36.8% had experienced 3 or 4 treatment failures. At Baseline the MADRS total score was classified as severe (total score ≥31) for 57.9% of participants and the mean MADRS total score was 31.7 (SD = 5.77) [40] (Table 1). At Baseline the mean CGI-S score was 4.3 (SD = 0.99) which falls into the ‘moderately ill’ range (score = 4).

Two psilocybin administration sessions (Day 1) involved simultaneous administration, where two participants were administrated at the same time (in separate rooms) in each session.

### Safety results

Seventeen TEAEs were reported for 12 participants (63.2%), none of which were considered serious, or led to study withdrawal (Table 2). Eleven of these TEAEs, which were experienced in nine participants (47.4%), had an onset of Day 1 (psilocybin administration day), and eight resolved on the same day. Of the six TEAEs experienced after Day 1 in four participants (21.1%), all but two resolved on the same day of onset. The most common TEAE reported was headache (six events; six participants [31.6%]) which had an onset of Day 1 (*n* = 4) or Day 2 (*n* = 2) which were predominantly transient, reported as mild and moderate in five and one participants, respectively, and resolved on their own or with standard medication (paracetamol and ibuprofen) on the same day or day after. Three participants had a TEAE of blood pressure increased which were considered related or possibly related to study treatment, two were severe and treated with clonidine. One of these patients experienced chest heaviness and headache (also relieved by clonidine). Unfortunately, we cannot give an accurate measure of the blood pressure elevation that undoubtedly occurred, because the finger cuff system was not accurately calibrated. There were no specific parameters for administering clonidine and it was down to investigator discretion. All TEAEs resolved before the end of the study (Week 3), and none had a duration exceeding seven days.

**Table 2:** Treatment-emergent adverse events reported from Day 1 to Week 3 (safety analysis set).

	COMP360 25 mg + SSRI (*N* = 19)	
	*n* (%)	Events
Overall		
Any TEAE	12 (63.2)	17
Any severe TEAE	2 (10.5)	2
Any serious TEAE	0	0
A. Day 1		
Any TEAE	9 (47.4)	11
Any severe TEAE	2 (10.5)	2
TEAE		
Headache	4 (21.1)	4
Blood pressure increased	3 (15.8)	3
Dizziness	1 (5.3)	1
Dry mouth	1 (5.3)	1
Fall	1 (5.3)	1
Skin abrasion	1 (5.3)	1
B. After Day 1 up to Week 3		
Any TEAE	4 (21.1)	6
Any severe TEAE	0	0
TEAEs		
Headache	2 (10.5)	2
Depression	1 (5.3)	1
Diarrhea	1 (5.3)	1
Palpitations	1 (5.3)	1
Vertigo	1 (5.3)	1

TEAEs coded using MedDRA version 23.0.

*MedDRA* Medical Dictionary for Regulatory Activities, *SSRI* selective serotonin reuptake inhibitor, *TEAE* treatment-emergent adverse event.

There were no reports of active suicidal ideation with plan or intent to act, attempts, or self-injurious behavior at Baseline or during the study and follow-up period (Supplementary Table S2). During the follow-up period, one participant who was disappointed by their lack of response and quality of psychedelic experience invoked concern regarding their mental state during a conversation with their non-study therapist, which prompted the therapist to initiate an involuntary assessment of the participant at a local emergency department. After evaluation by a psychiatrist in the emergency department, the participant was deemed to not be a danger to themselves and not in need of hospital admission. An evaluation by a study psychiatrist shortly after the release from the emergency department arrived at the same conclusion.

There were no clinically meaningful shifts from Baseline in clinical laboratory tests. No participant had treatment-emergent clinically important ECG values or increases in QTcF interval from Baseline.

### Primary and secondary efficacy results

**Fig. 1:**
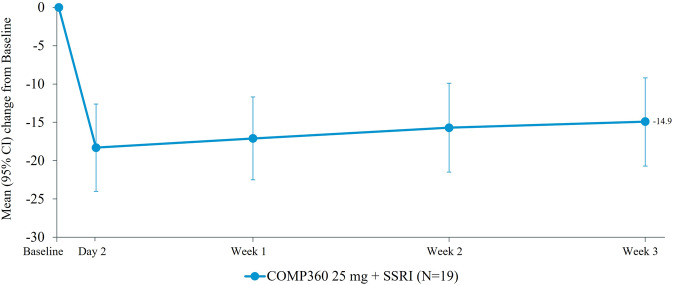
Change from baseline in MADRS total score (full analysis set). CI confidence interval, MADRS Montgomery-Åsberg depression rating scale, SD standard deviation, SSRI selective serotonin reuptake inhibitor. Note: Baseline mean (SD): COMP360 25 mg + SSRI = 31.7 (5.77).

The mean MADRS total score at Week 3 was 16.8 (95% confidence interval [CI], 11.2–22.4) which equated to a clinically meaningful mean change from Baseline of −14.9 (95% CI, −20.7 to −9.2). This improvement was apparent at Day 2 and maintained throughout the three-week follow-up (Fig. 1). An additional post-hoc analysis was performed to analyze the change from Baseline to Week 3 for individual items from the MADRS to indicate whether certain symptoms of depression were more subject to change following treatment with psilocybin. A reduction in every item in the MADRS was apparent from Baseline to Week 3 (Supplementary Fig. S2).

Response (≥50% reduction from Baseline MADRS total score) at Week 3 was evident in eight (42.1%) participants and was sustained throughout the follow-up period for 63.2% at Day 2, and 57.9% at Weeks 1 and 2. The same 42.1% of participants who were responders at Week 3 were also remitters (MADRS total score of ≤10) at Week 3. Remission rates remained high throughout the follow-up period for 52.6% at Day 2, 47.4% at Week 1, and 42.1% at Week 2 (Supplementary Table S3; Supplementary Fig. S3).

**Fig. 2:**
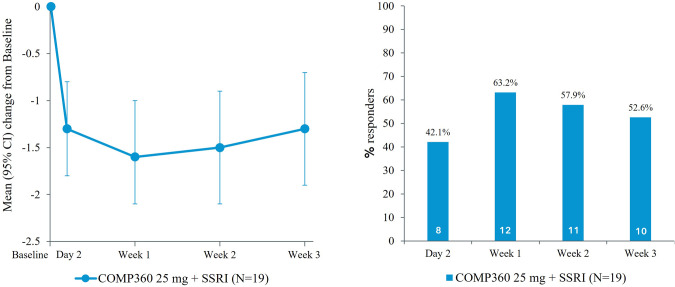
Change from baseline in CGI-S score and proportion of CGI-S responders over time (full analysis set). CGI-S Clinical Global Impressions – Severity, CI confidence interval, SSRI selective serotonin reuptake inhibitor. Number of CGI-S responders stated in bars. Note: Mean change from Baseline CGI-S score was −1.3 at Week 3.

The mean CGI-S score at Week 3 was 2.9 (SD = 1.84) which equates to a −1.3 (SD = 1.29) change from Baseline. Proportion of responders on the CGI-S were 42.1% at Day 2, 63.2% at Week 1, 57.9% at Week 2, and 52.6% at Week 3 [Fig. 2].

The results from per-protocol analysis of the primary end point were consistent with the full analysis set.

### Exploratory results

Improvements in quality of life, wellbeing, anxiety, affect, and self- and clinician-reported depression were evident at all time points following psilocybin administration.

Participants’ EQ-5D-3L mean total score at Baseline was 0.51 (SD = 0.333) and improved by an average of 0.18 (SD = 0.310) by Week 3. The EQ-VAS mean score at Baseline was 49.0 (SD = 17.45) and improved by an average of 13.7 (SD = 18.09) by Week 3. The GAD-7 mean total score at Baseline was 12.9 (SD = 5.94) and decreased by an average of 4.1 (SD = 4.90) by Week 3, which is considered a clinically meaningful improvement in anxiety symptoms [41]. The QIDS-SR-16 mean total score at Baseline was 16.5 (SD = 5.04), indicating severe depression, and decreased by an average of 6.9 (SD = 6.05) by Week 3 where the mean total score indicated mild depression. An overall improvement was observed for the PANAS. The mean Baseline score for positive affect was 19.3 (SD = 7.22) and increased by an average of 8.1 (SD = 10.58) by Day 2, and mean Baseline score for negative affect was 24.3 (SD = 10.26) and decreased by an average of 7.5 (SD = 6.79) by Day 2 (Supplementary Table S4).

Fourteen participants (73.7%) were considered at least minimally improved (CGI-I score ≤3) by clinicians from Baseline to Week 3 and seven participants (36.8%) were considered responders (score or 1 or 2) at Week 3. No participants were considered worsened based on the CGI-I (score ≤5) at Week 3. See Supplementary appendix for CGI-I summary of responders (Supplementary Table S5).

**Fig. 3:**
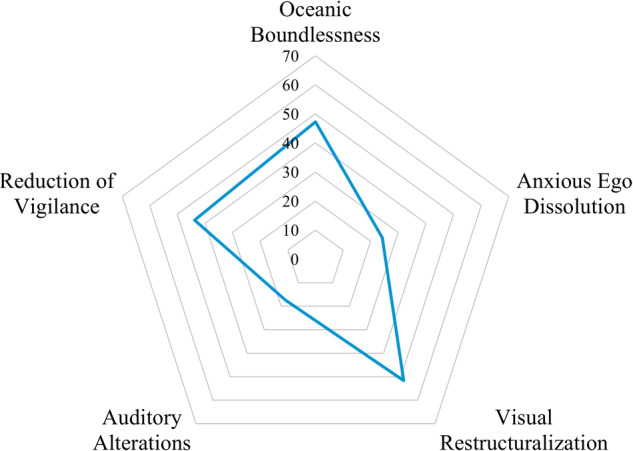
Summary of 5D-ASC Dimension Scores on Day 1 (full analysis set).

Alterations of consciousness on the 5D-ASC were observed in every dimension including visual restructuralization, oceanic boundlessness, reduction of vigilance, and to a lesser extent anxious ego dissolution and auditory alterations (Fig. 3; Supplementary Table S6).

## Discussion

In participants with TRD, a single administration of psilocybin 25 mg with psychological support adjunctive to an SSRI demonstrated a generally favorable safety profile and therapeutic efficacy across a range of clinician and self-rated measures. Contrary to speculation in prior literature [9, 11, 42], this does not support the hypothesis that adjunctive administration of psilocybin diminishes the antidepressant effects of psilocybin.

Most TEAEs were mild, short-lived, and resolved on the day of onset without medical intervention, occurring in just over half of participants. A TEAE of blood pressure increase occurred in three participants. Effects of psilocybin on heart rate and blood pressure have been reported in the literature before [43–46], but these specific instances were recorded with a system which may not have been properly calibrated and were not checked by conventional cuff auscultation. There were no serious TEAEs and no TEAE led to study withdrawal. Suicidal ideation or behavior measured by the C-SSRS did not increase during the study, and there were no clinically significant abnormal laboratory evaluations or ECGs. The resulting safety profile is comparable to that of psilocybin monotherapy in an open-label or controlled design [5, 47] and suggests that the safety of psilocybin in this context is not compromised when administered adjunct to an SSRI. Nevertheless, vigilance for idiosyncratic psychological reactions to treatment failure will continue to be necessary in following up patients in trials of the psychedelic experience.

There were meaningful improvements in depression severity from Baseline to Week 3, indicating that ongoing SSRI treatment did not inhibit the therapeutic potential of psilocybin. Improvements were evident across measures of depression assessed by an independent clinician (MADRS), investigator (CGI-S), and self-report (QIDS-SR-16) and persisted from Day 2 to the end of the study. Changes were apparent in every item of the MADRS with substantial improvement in the symptoms thought to be core to depression. Consistent with these findings, exploratory measures of quality of life, wellbeing, affect, and anxiety indicated improvements from Baseline across all timepoints.

Although the difference in study design limits formal comparison, these efficacy findings are similar to those from the largest phase IIb randomized controlled study of psilocybin in TRD (COMP 001) in which antidepressant withdrawal was required prior to psilocybin administration [5, 6]; in fact, the response and remission rates were higher in the current study (42% response and remission rates at Week 3 verses 37% and 29%, respectively). These response rates are also substantially higher than those reported for treatment-resistant patients studied systematically in STAR*D [48]. This indicates that antidepressant drug withdrawal is not a prerequisite for a therapeutic response to psilocybin in this patient group, despite most previous trials including withdrawal as a standard procedure [19, 20]. Future research should examine whether the outcomes of psilocybin as a monotherapy or as an adjunct to SSRIs remain comparable in a longer term follow up.

A single study has previously examined the psychedelic effect of psilocybin co-administered with an SSRI, using controlled, randomized, cross-over design in healthy volunteers [21]. Treatment with escitalopram for two weeks did not modify the magnitude of the positive dimensions of the 5D-ASC produced by a single 25 mg dose, when compared to placebo. However, escitalopram did reduce negative drug experiences, anxiety, and adverse cardiovascular effects. The acute psychedelic effects captured by the 5D-ASC in healthy volunteers [21] aligned with the findings in the current study, where the greatest alterations were in the “visual restructuralization”, “oceanic boundlessness”, and “reduction of vigilance” dimensions. The 5D-ASC outcome in the present study is also consistent with a meta-analysis of studies of psilocybin in healthy volunteers and COMP 001 where participants with TRD were withdrawn from antidepressant drugs [5, 49]. Taken together, these findings suggest that the pharmacological interactions necessary for psilocybin to take effect are not meaningfully compromised by SSRIs [9].

The acute subjective effects resulting from psilocybin and linked to its agonist activity at 5-HT2A/C receptors are suggested to be critical to therapeutic response [49, 50]. Despite the open-label design of the present study, this replication of the outcome of the 5D-ASC would likely not be apparent if 5-HT2A/C receptors were meaningfully downregulated by chronic SSRI treatment. It is possible that the anticipated reduction of 5-HT2A/C receptors in the post-synaptic space following treatment with SSRIs has a smaller effect on the psychedelic experience than theories suggest [9, 51]. This may be explained by the lower binding affinity of psilocybin at 5-HT2A compared to other psychedelics such as LSD [18], reducing the proposed impact of the downregulation caused by SSRIs. It is also possible that unknown mechanisms downstream of 5-HT2A/C receptor signaling can compensate for any effects that chronic SSRI treatment might have on the psychedelic response.

The freedom to administer psilocybin as a treatment adjunctive to an ongoing SSRI has important implications for clinical practice. Allowing continuation of antidepressant drugs would increase treatment accessibility for those who prefer to stay on their current medication and reduce the potential negative impact of antidepressant drug withdrawal or tapering. The present study was limited by its small size, open-label design and absence of a comparator, the lack of a demographically diverse sample, and the exclusion of participants with high suicide risk or current hospitalization. It is possible that the open-label design influenced participant bias and expectations towards the study treatment, and definitive comparisons with studies that included a withdrawal from SSRIs is not necessarily valid. The findings will need to be confirmed in a larger, double-blind, comparator-controlled study.

This study found that a single, open-label administration of psilocybin 25 mg led to an acceptable experience for participants with TRD when administrated adjunct to an SSRI and supports further development of psilocybin with psychological support for people with TRD. This study demonstrated the feasibility of simultaneous psilocybin administration to multiple participants as previously reported [1, 52], provided that adequate support is available. This design could reduce resource requirements for administration and provide a more accessible option for future research or in clinical practice. These encouraging results suggest that further investigation of psilocybin adjunct to antidepressant drugs would be valuable, especially in cases where antidepressant drug withdrawal may not be desirable.

## Supplementary information

Supplementary Appendix
