# Supplementary material for: Psilocybin for treatment resistant depression in patients taking a concomitant SSRI medication
Source: Neuropsychopharmacology. 2023 Jul 13;48(10):1492–9. doi: 10.1038/s41386-023-01648-7 (PMC10425429; doi:10.1038/s41386-023-01648-7)
Supplement: Supplementary file 1 — Supplementary Appendix [file 41386_2023_1648_MOESM1_ESM.docx]

Supplementary Appendix

**Supplement to:** Goodwin G. M. et al. Psilocybin for Treatment Resistant Depression in Patients Taking a Concomitant SSRI Medication

Table of Contents

[Supplementary Methods 3](#_Toc137537635)

[Eligibility Criteria 3](#_Toc137537636)

[Inclusion Criteria 3](#_Toc137537637)

[Exclusion Criteria 4](#_Toc137537638)

[Table S1: Schedule of Assessments 7](#_Toc137537639)

[Figure S1: CONSORT Flowchart 9](#_Toc137537640)

[Supplementary Results 10](#_Toc137537641)

[Table S2: C-SSRS Shift from Baseline to Worst Post-Baseline Score from Day 1 to Week 3 10](#_Toc137537642)

[Table S3: Primary and Secondary Outcomes (Full Analysis Set) 11](#_Toc137537643)

[Figure S2: Change from Baseline to Week 3 in MADRS Individual Items (Full Analysis Set) 12](#_Toc137537644)

[Figure S3: Proportion of MADRS Responders and Remitters (Full Analysis Set) 14](#_Toc137537645)

[Table S4: Exploratory Endpoints (Full Analysis Set) 15](#_Toc137537646)

[Table S5: CGI-I Summary of Responders (Full Analysis Set) 16](#_Toc137537647)

[Table S6: Summary of 5D-ASC Dimension Scores on Day 1 (Full Analysis Set) 17](#_Toc137537648)

[References 18](#_Toc137537649)

# Supplementary Methods

## Eligibility Criteria

### Inclusion Criteria

Participants meeting all the following inclusion criteria at Screening were considered for admission into the study.

1. Signed Informed Consent Form (ICF).
2. 18 years of age or older at Screening.
3. At least moderate major depressive disorder (MDD) (single or recurrent episode as informed by Diagnostic and Statistical Manual of Mental Disorders, Fifth Edition (DSM-5); if single episode duration of ≥3 months and ≤2 years) based on medical records, clinical assessment, and documented completion of the Mini International Neuropsychiatric Interview (MINI) version 7.0.2.
4. 17 item, Hamilton Depression Rating Scale total score ≥18 at Screening and Baseline.
5. Currently receiving treatment with a selective serotonin reuptake inhibitor (SSRI) (fluoxetine, fluvoxamine, sertraline, paroxetine, citalopram, vilazodone, vortioxetine, or escitalopram) at, or above, a minimum locally approved therapeutic dose for at least 6 weeks before Screening and Baseline. Dose changes within the adequate range were acceptable. To be defined as an adequate study, adherence of at least 75% was needed based on participant estimate of percentage of doses that were taken.
6. Failure to respond to an adequate dose and duration of two, three, or four pharmacological treatments for the current episode as determined through the Massachusetts General Hospital Antidepressant Treatment Response Questionnaire (MGH-ATRQ) and using the supplementary advice on additional antidepressants not included in MGH-ATRQ. Augmentation with an add on treatment counted as a second treatment, provided it was approved for the adjunctive treatment of MDD in that country.
7. McLean Screening Instrument for Borderline Personality Disorder (MSI-BPD) <7 at Screening.
8. Ability to complete all protocol required assessment tools without any assistance or alteration to the copyrighted assessments, and to comply with all study visits.

### Exclusion Criteria

Participants meeting any of the following exclusion criteria at Screening were not enrolled into the study.

*Psychiatric Exclusion Criteria:*

1. Current or past history of schizophrenia, psychotic disorder (unless substance induced or due to a medical condition), bipolar disorder, delusional disorder, paranoid personality disorder, schizoaffective disorder, or borderline personality disorder, as assessed by medical history, MSI-BPD and a structured clinical interview (MINI version 7.0.2).
2. Prior electroconvulsive therapy and/or ketamine for current episode.
3. Ongoing use of an antidepressant medication, including augmentation or combination therapies, other than a single SSRI at Screening and Baseline.
4. Current psychological therapies that would not remain stable within 21 days of the COMP360 administration session. Psychological therapies cannot be initiated within 21 days of Baseline.
5. Current (within the last year) alcohol or substance use disorder as informed by DSM-5 (diagnosed by MINI version 7.0.2) at Screening.
6. Significant suicide risk as defined by (1) suicidal ideation as endorsed on items 4 or 5 on the Columbia-Suicide Severity Rating Scale (C-SSRS) within the past year, at Screening or at Baseline, or; (2) suicidal behaviours within the past year, or; (3) clinical assessment of significant suicidal risk during participant interview.
7. Depression secondary to other severe medical conditions according to clinicians’ judgement.
8. Other personal circumstances and behaviour judged to be incompatible with establishment of rapport or safe exposure to psilocybin, including exposure to psilocybin within the past year and use of psychedelics, such as ayahuasca, during the current depressive episode.

*General Medical Exclusion Criteria:*

1. Females who are pregnant, nursing, or planning a pregnancy. Male and female participants who engaged in sexual intercourse which could result in pregnancy, had to agree to use a highly effective contraceptive method throughout their participation in the study. Females of childbearing potential must have had a negative urine pregnancy test at Screening and Baseline.
2. Cardiovascular conditions: recent stroke (<1 year from signing of ICF), recent myocardial infarction (<1 year from signing of ICF), hypertension (blood pressure >140/90 mmHg), or clinically significant arrhythmia within 1 year of signing the ICF.
3. Uncontrolled or insulin-dependent diabetes.
4. Seizure disorder.
5. Positive urine drug screen for illicit drugs or drugs of abuse at Screening and Baseline. Any positive urine drug test was reviewed with participants to determine the pattern of use and eligibility will be determined at the investigator’s discretion in conjunction with the medical monitor.
6. Current enrolment in any investigational drug or device study or participation in such within 30 days prior to Screening.
7. Current enrolment in another clinical study of an investigational medical or participation in such within 30 days of Screening.
8. Abnormal and clinically significant results on the physical examination, vital signs, electrocardiogram (ECG), or laboratory tests at Screening.
9. Any other clinically significant cardiovascular, pulmonary, gastrointestinal, hepatic, renal or any other major concurrent illness that, in the opinion of the investigator, may have interfered with the interpretation of the study results or constitute a health risk for the participant if he/she took part in the study.

## Table S1: Schedule of Assessments

|  |  | **3 weeks**  **prior to**  **Baseline** |  |  | **Time post COMP360 administration** | | | |
| --- | --- | --- | --- | --- | --- | --- | --- | --- |
|  | **Screening** | **Screening Period^1^** | **Baseline**  **(Day -1)** | **COMP360 Administration**  **(Day 1)** | **Day 2** | **Week 1**  **Day 8** | **Week 2**  **Day 15** | **Week 3**  **Day 22**  **(EoS/ET)** |
| **Visit** | **1** | **1a, 1b, 1c** | **2** | **3** | **4** | **5** | **6** | **7** |
| **Allowable Window** |  | **Weekly** |  | **+ ≤7 days** | **None** | **±1 day** | **±1 day** | **± 1 day** |
| **Clinical Assessments and Procedures** | | | | | | | | |
| Informed consent | ✓ |  |  |  |  |  |  |  |
| Medical history | ✓ |  | ✓ |  |  |  |  |  |
| Inclusion/Exclusion criteria | ✓ |  | ✓ |  |  |  |  |  |
| MINI 7.0.2 | ✓ |  |  |  |  |  |  |  |
| HAM-D-17 | ✓ |  | ✓ |  |  |  |  |  |
| MGH-ATRQ | ✓ |  |  |  |  |  |  |  |
| C-SSRS^2^ | ✓ | ✓ | ✓ | ✓ | ✓ | ✓ | ✓ | ✓ |
| Vital signs | ✓ |  | ✓ | ✓ | ✓ |  |  |  |
| Physical examination, including weight and height | ✓ |  |  |  |  |  |  |  |
| 12-lead ECG | ✓ |  |  |  | ✓ |  |  |  |
| Clinical laboratory tests | ✓ |  |  |  | ✓ |  |  | ✓ |
| Urinalysis | ✓ |  | ✓ |  | ✓ |  |  |  |
| Urine drug screen | ✓ |  | ✓ |  |  |  |  |  |
| Urine pregnancy test^3^ | ✓ |  | ✓ |  |  |  |  |  |
| Documentation of contraceptive method to be used^4^ | ✓ |  |  |  |  |  |  |  |
| CGI-I |  |  |  |  | ✓ | ✓ | ✓ | ✓ |
| CGI-S |  |  | ✓ |  | ✓ | ✓ | ✓ | ✓ |
| Preparation |  | ✓ | ✓ |  |  |  |  |  |
| COMP360 administration |  |  |  | ✓ |  |  |  |  |
| Integration |  |  |  |  | ✓ | ✓ |  |  |
| Prior/Concomitant medications | ✓ | ✓ | ✓ | ✓ | ✓ | ✓ | ✓ | ✓ |
| AE/SAEs | ✓ | ✓ | ✓ | ✓ | ✓ | ✓ | ✓ | ✓ |
| **Participant Completed Assessments** | | | | | | | | |
| MSI-BPD | ✓ |  |  |  |  |  |  |  |
| QIDS-SR-16 | ✓ |  | ✓ | ✓ | ✓ | ✓ | ✓ | ✓ |
| EQ-5D-3L |  |  | ✓ |  |  | ✓ | ✓ | ✓ |
| GAD-7 |  |  | ✓ |  | ✓ | ✓ | ✓ | ✓ |
| PANAS |  |  | ✓ |  | ✓ |  |  |  |
| 5D-ASC |  |  |  | ✓^5^ |  |  |  |  |
| **Remote Rater Assessments** | | | | | | | | |
| MADRS^6^ |  |  | ✓ |  | ✓ | ✓ | ✓ | ✓ |

5D-ASC=Five Dimension Altered States of Consciousness; AE=adverse event; CGI-I=Clinical Global Impression–Improvement; CGI-S=Clinical Global Impression–Severity; C‑SSRS=Columbia-Suicide Severity Rating Scale; ECG=electrocardiogram; EoS=end of study; EQ-5D-3L=EQ-5D-3 level; ET=early termination; GAD-7=Generalized Anxiety Disorder 7-item Scale; HAM-D-17=Hamilton Depression Rating Scale (17-item); MINI 7.0.2=Mini International Neuropsychiatric Interview, version 7.0.2; MADRS=Montgomery-Åsberg Depression Rating Scale; MSIBPD=McLean Screening Instrument for Borderline Personality Disorder; PANAS=Positive and Negative Affect Schedule; QIDS-SR-16=Quick Inventory of Depressive Symptomatology – Self Rated - 16 item; SAE= serious adverse event.

^1^ Screening (Visit 1a) was performed approximately 3 weeks prior to the Baseline visit (Visit 2, Day -1).

^2^ The “Last 12 months” version was administered at Screening and the “Since Last Visit” version was administered at all other visits.

^3^ For females of child-bearing potential only.

^4^ For females of childbearing potential and all males; site was to document method of contraception agreed to be used by each participant.

^5^ Was to be administered immediately after the COMP360 administration session.

^6^ On site clinic visits; visits allowed remotely had the MADRS performed by a blinded telephone rater.

All visits conducted remotely were reported as a protocol deviation.

## Figure S1: CONSORT Flowchart


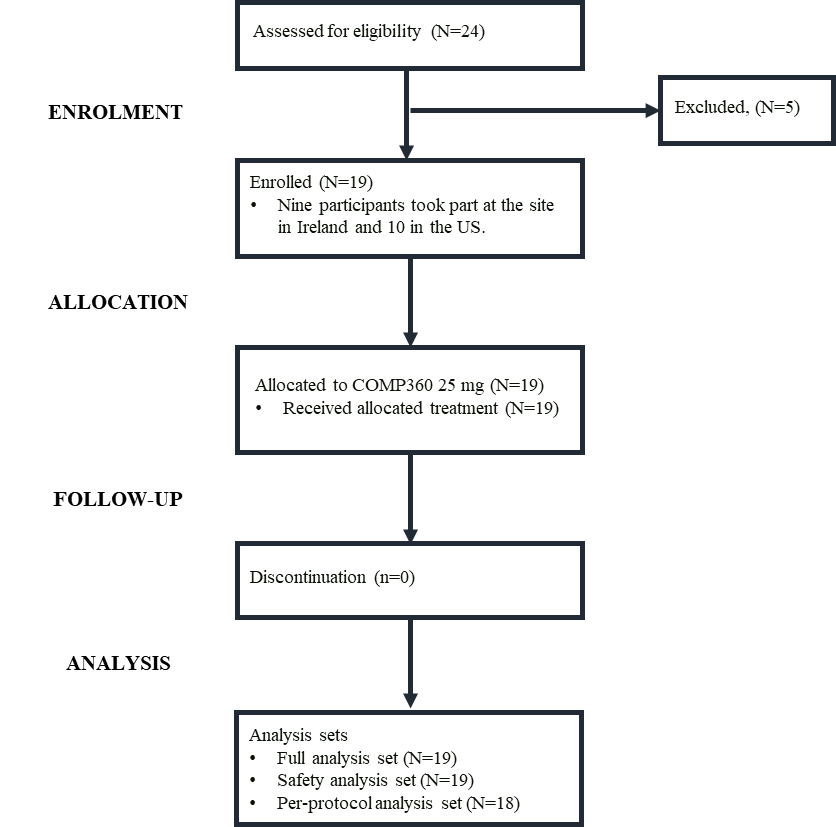


All exclusions were due to satisfying inclusion/exclusion criteria during screening.

One participant had an important protocol deviation of violating exclusion criteria 2 which resulted in their exclusion from the per-protocol analysis set.

The trial ended when target recruitment was met.

# Supplementary Results

## Table S2: C-SSRS Shift from Baseline to Worst Post-Baseline Score from Day 1 to Week 3

|  | | **Worst Post-Baseline C-SSRS Score** | | | | | | | |
| --- | --- | --- | --- | --- | --- | --- | --- | --- | --- |
| **Treatment Group** | **C-SSRS Baseline Score** | **0** | **1** | **2** | **3** | **4** | **5** | **Missing** | **Total** |
| COMP360 25 mg + SSRI (N=19) | 0 | 12 (63.2) | 1 (5.3) | 1 (5.3) | 0 | 0 | 0 | 0 | 14 (73.7) |
|  | 1 | 3 (15.8) | 2 (10.5) | 0 | 0 | 0 | 0 | 0 | 5 (26.3) |
|  | 2 | 0 | 0 | 0 | 0 | 0 | 0 | 0 | 0 |
|  | 3 | 0 | 0 | 0 | 0 | 0 | 0 | 0 | 0 |
|  | 4 | 0 | 0 | 0 | 0 | 0 | 0 | 0 | 0 |
|  | 5 | 0 | 0 | 0 | 0 | 0 | 0 | 0 | 0 |
|  | Missing | 0 | 0 | 0 | 0 | 0 | 0 | 0 | 0 |
|  | Total | 15 (78.9) | 3 (15.8) | 1 (5.3) | 0 | 0 | 0 | 0 | 19 (100.0) |

C-SSRS=Columbia Suicidality Severity Rating Scale.

C-SSRS scoring: 0=no suicidal ideation items endorsed; 1=wish to be dead; 2=nonspecific active suicidal thoughts; 3=active suicidal ideation with any methods (no plan) without intent to act; 4=active suicidal ideation with some intent to act, without specific plan; 5=active suicidal ideation with specific plan and intent.

## Table S3: Primary and Secondary Outcomes (Full Analysis Set)

|  | **COMP360 25 mg + SSRI**  **(N=19)** |
| --- | --- |
| **Primary efficacy endpoint** |  |
| MADRS total score change from Baseline at Week 3 |  |
| Mean (SD)*d* | -14.9 (11.97) *-1.249* |
| 95% CI | -20.7, -9.2 |
| Median (min, max) | -15.0 (-32, 5) |
| **Secondary efficacy endpoint** |  |
| MADRS proportion of responders at Week 3, n (%) | 8 (42.1%) |
| MADRS proportion of remitters at Week 3, n (%) | 8 (42.1%) |
| CGI-S score change from Baseline at Week 3, Mean (SD) *d* | -1.3 (1.29) *-1.017* |
| CGI-S proportion of responders at Week 3 | 10 (52.6) |

CGI-S=Clinical Global Impression– Severity; CI=confidence interval; *d* =Cohen’s d; MADRS=Montgomery‑Åsberg Depression Rating Scale; Max= maximum; Min= minimum; SD= standard deviation; SSRI= selective serotonin reuptake inhibitor.

##
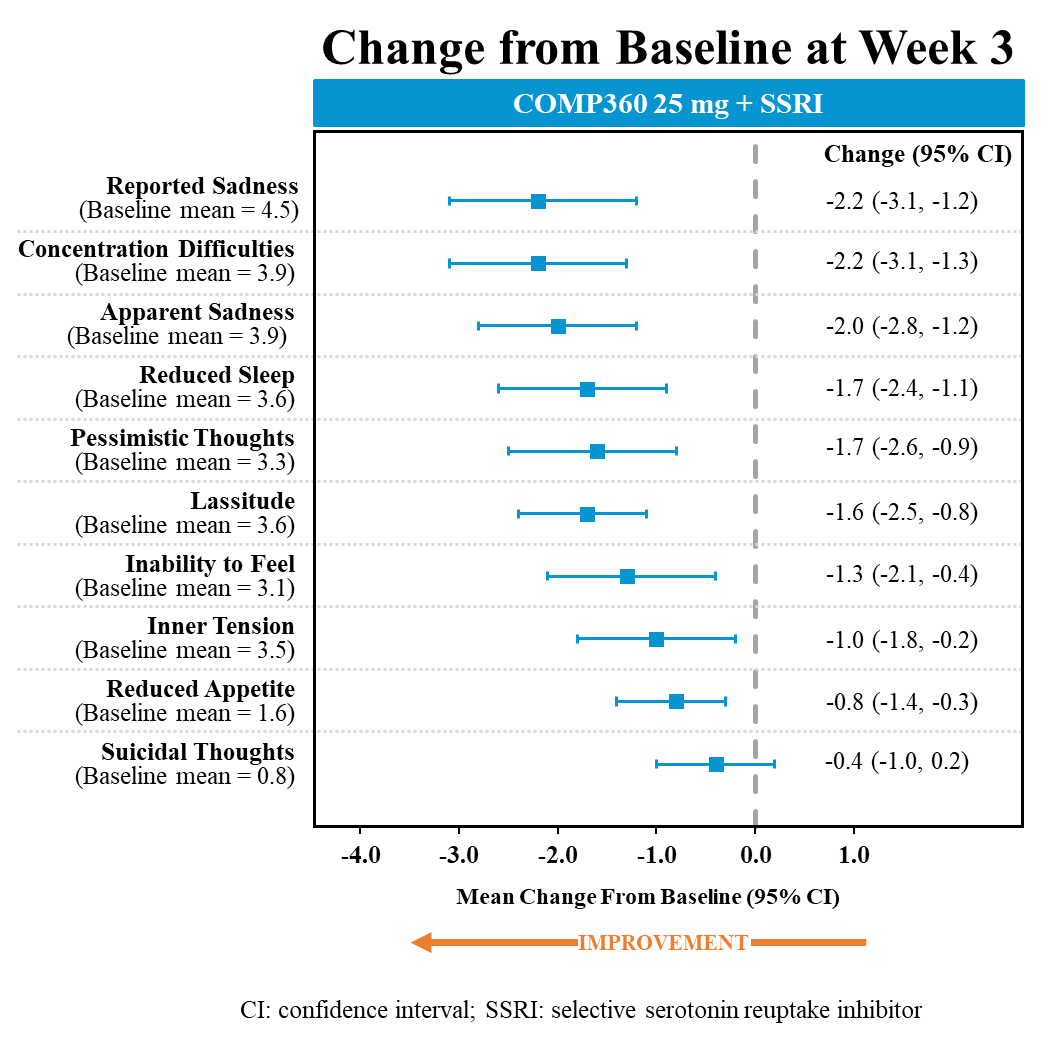
Figure S2: Change from Baseline to Week 3 in MADRS Individual Items (Full Analysis Set)

CI=confidence interval; MADRS=Montgomery-Åsberg depression rating scale; SSRI=selective serotonin reuptake inhibitor.

The single-item analysis revealed changes in every item of the MADRS with substantial improvement in the symptoms thought to be core to depression (apparent sadness, reported sadness, lassitude, and inability to feel), in addition to reduced sleep, concentration difficulties, and pessimistic thoughts. The greatest reduction was evident for apparent sadness, reported sadness, and concentration difficulties. This observation is comparable to findings in an esketamine trial [1] and suggests that these symptoms are fundamental to the mechanism of antidepressant effect for both esketamine and psilocybin. The reduction of symptoms concerning anhedonia (apparent sadness, reported sadness, lassitude, inability to feel, concentration difficulties; [2,3]) is particularly promising since such symptoms have been associated with greater severity of depression and poorer outcome after treatment with standard of care [4,5].

##
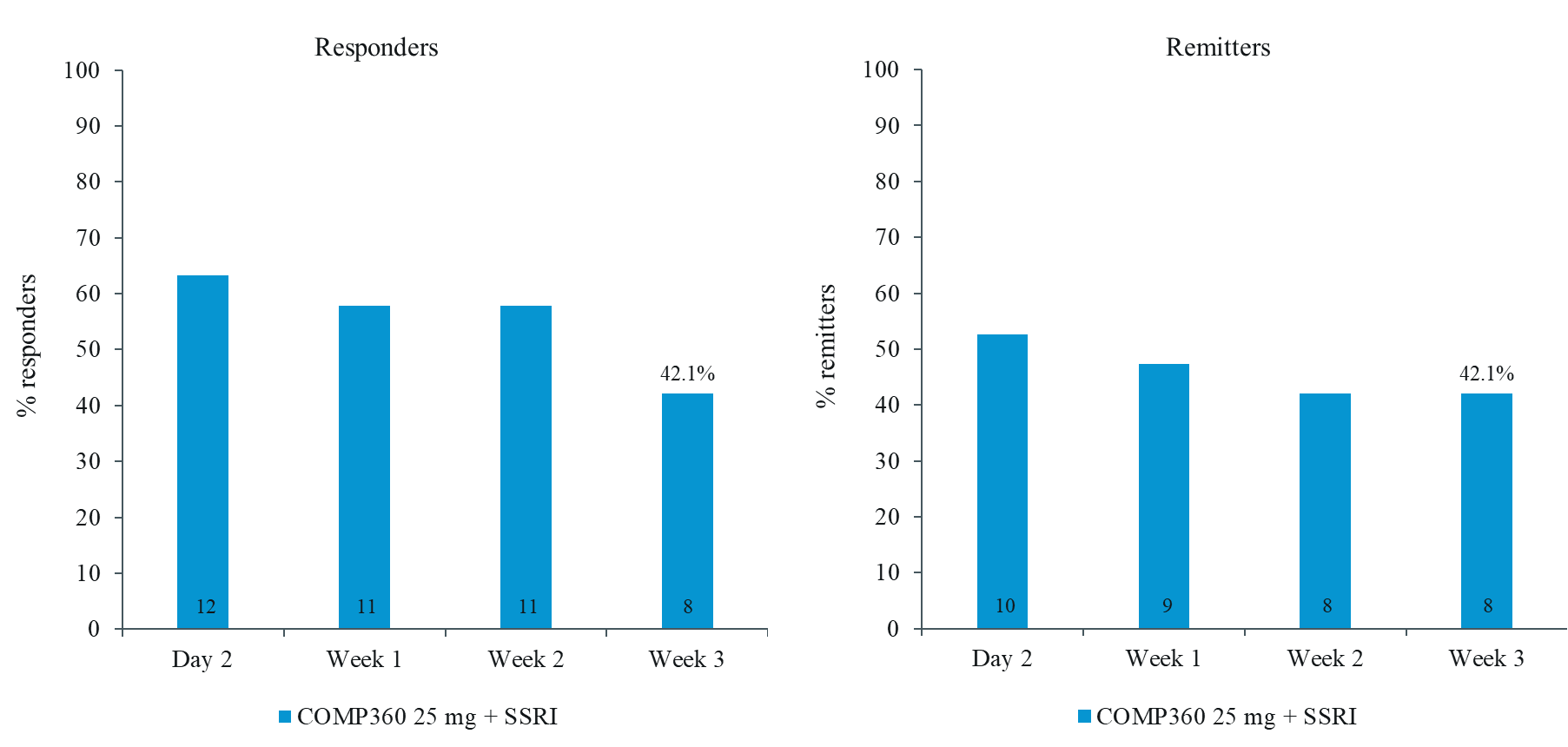
Figure S3: Proportion of MADRS Responders and Remitters (Full Analysis Set)

MADRS=Montgomery-Åsberg Depression Rating Scale; SSRI=selective serotonin reuptake inhibitor.

Note: Number of MADRS responders/remitters is stated in the bars.

## Table S4: Exploratory Endpoints (Full Analysis Set)

|  | **COMP360 25 mg + SSRI**  **(N=19)** |
| --- | --- |
| **Exploratory endpoints** | **Mean (95% CI)** |
| EQ-5D-3L total score change from Baseline to Week 3 | 0.18 (0.03, 0.33) |
| EQ-VAS total score change from Baseline to Week 3 | 13.7 (5.0, 22.5) |
| GAD-7 total score change from Baseline to Week 3 | -4.1 (-6.5, -1.7) |
| QIDS-SR-16 total score change from Baseline to Week 3 | -6.9 (-9.9, -4.0) |
| PANAS Positive Affect score change from Baseline to Day 2 | 8.1 (2.9, 13.4) |
| PANAS Negative Affect score change from Baseline to Day 2 | -7.5 (-10.9, -4.1) |

CI=confidence interval; EQ-5D-3L=EQ-5D-3 level; GAD-7=Generalized Anxiety Disorder 7-item; N=number of participants; PANAS=Positive and Negative Affect Schedule; QIDS-SR-16=Quick Inventory of Depressive Symptomatology 16-item; SSRI=selective serotonin reuptake inhibitor.

## Table S5: CGI-I Summary of Responders (Full Analysis Set)

|  | **COMP360 25 mg + SSRI**  **(N=19)** | | | |
| --- | --- | --- | --- | --- |
|  | **Day 2** | **Week 1** | **Week 2** | **Week 3** |
| n (%) | 11 (57.9) | 12 (63.2) | 8 (42.1) | 7 (36.8) |
| 95% CI | 35.7, 80.1 | 41.5, 84.8 | 19.9, 64.3 | 15.2, 58.5 |

CI=confidence interval; CGI-I=Clinical Global Impression–Improvement; SSRI=selective serotonin reuptake inhibitor.

Note: Responders were defined as participants that respond either 1 (‘very much improved’) or 2 (‘much improved’).

## Table S6: Summary of 5D-ASC Dimension Scores on Day 1 (Full Analysis Set)

| **Dimension**  Statistic | **COMP360 25 mg + SSRI**  **(N=19)** |
| --- | --- |
| **Oceanic boundlessness** |  |
| Mean (SD) | 47.32 (30.381) |
| Median (min, max) | 46.70 (0.0, 95.9) |
| **Anxious ego dissolution** |  |
| Mean (SD) | 24.17 (20.267) |
| Median (min, max) | 20.20 (1.2, 71.3) |
| **Visual restructuralization** |  |
| Mean (SD) | 51.72 (27.187) |
| Median (min, max) | 58.20 (0.0, 86.3) |
| **Auditory alterations** |  |
| Mean (SD) | 17.43 (20.724) |
| Median (min, max) | 9.20 (0.0, 65.3) |
| **Reduction of vigilance** |  |
| Mean (SD) | 43.70 (24.361) |
| Median (min, max) | 48.50 (5.9, 85.7) |

5D-ASC=Five-Dimensional – Altered States of Consciousness; Max=maximum; Min=minimum; SD=standard Deviation; SSRI=selective serotonin reuptake inhibitor.

## References

1 Floden L, Hudgens S, Jamieson C, Popova V, Drevets WC, Cooper K, et al. Evaluation of Individual Items of the Patient Health Questionnaire (PHQ-9) and Montgomery-Asberg Depression Rating Scale (MADRS) in Adults with Treatment-Resistant Depression Treated with Esketamine Nasal Spray Combined with a New Oral Antidepressant. CNS Drugs. 2022;36(6):649-58.

2 Borentain S, Gogate J, Williamson D, Carmody T, Trivedi M, Jamieson C, et al. Montgomery-Åsberg Depression Rating Scale factors in treatment-resistant depression at onset of treatment: Derivation, replication, and change over time during treatment with esketamine. International Journal of Methods in Psychiatric Research.n/a(n/a):e1927.

3 Cao B, Park C, Subramaniapillai M, Lee Y, Iacobucci M, Mansur RB, et al. The Efficacy of Vortioxetine on Anhedonia in Patients With Major Depressive Disorder. Front Psychiatry. 2019;10:17.

4 Uher R, Perlis RH, Henigsberg N, Zobel A, Rietschel M, Mors O, et al. Depression symptom dimensions as predictors of antidepressant treatment outcome: replicable evidence for interest-activity symptoms. Psychological Medicine. 2012;42(5):967-80.

5 Tolentino JC, Schmidt SL. DSM-5 Criteria and Depression Severity: Implications for Clinical Practice. Frontiers in Psychiatry. 2018;9.
